# Spasmolytic and Antibacterial Activity of Two* Citrus sinensis* Osbeck Varieties Cultivated in Mexico

**DOI:** 10.1155/2017/3960837

**Published:** 2017-03-05

**Authors:** Amanda Sánchez-Recillas, Ana Ly Arroyo-Herrera, Jesús Alfredo Araujo-León, Emanuel Hernández Núñez, Rolffy Ortiz Andrade

**Affiliations:** ^1^Laboratorio de Farmacología, Facultad de Química, Universidad Autónoma de Yucatán, Mérida, YUC, Mexico; ^2^Laboratorio de Cromatografía, Facultad de Química, Universidad Autónoma de Yucatán, Mérida, YUC, Mexico; ^3^CONACYT, Departamento de Recursos del Mar, CINVESTAV-Unidad Mérida, Mérida, YUC, Mexico

## Abstract

Antibacterial activity on ATCC strains of* Escherichia coli*,* Salmonella enterica*,* Salmonella enteritidis,* and* Salmonella choleraesuis* and spasmolytic effect on contraction on rat ileum trips were determinate. Eight organic extracts (hexanic and methanolic) of albedo (mesocarp) and flavedo (pericarp) of two varieties (Valencian and National) of* Citrus sinensis* (L.) Osbeck of Yucatán, México, were studied. Additionally, chromatographic fingerprints were obtained and correlated with their pharmacological effects. MAN, MAV, and HFN extract caused inhibition against* S. choleraesuis *(MIC: 1000 *µ*g/mL) and* S. enteritidis* (MIC: 1000 *µ*g/mL). Regarding the spasmolytic effect, the Valencian extracts variety was more efficient on spontaneous contraction, HAV (*E*_max_ = 51.98 ± 1.98%), MAV (*E*_max_ = 35.98 ± 1.42%), HFV (*E*_max_ = 68.91 ± 4.14%), and MFV (*E*_max_ = 51.28 ± 2.59%), versus National variety, HAN (*E*_max_ = 43.80 ± 6.32%), MAN (*E*_max_ = 14.62 ± 1.69%), HFN (*E*_max_ = 64.87 ± 3.04%), and MFN (*E*_max_ = 31.01 ± 3.92%). Chromatographic fingerprints of HFV and HFN were found to have some similar signals that belong to monoterpenes, whereas for HAN and HAV similar signals were found belonging to fatty acids and triterpenoids. Methanolic extracts showed signals of (1) furfural, (2) furfural acetone (3) furfuraldehyde and (4) *β*–sitosterol compounds. Flavedo portion of* C. sinensis* possessed spasmolytic effect on rat ileum strips and antibacterial activity against* Salmonella* strains. This species is source for obtaining bioactive compounds with therapeutic potential in the treatment of infectious diarrhea.

## 1. Introduction

Diarrhea is the passage of three or more loose or liquid stools per day, or more frequently than normal for the individual. It is usually a symptom of gastrointestinal infection, which can be caused by a variety of bacterial, viral, and parasitic organisms [1]. Severe diarrhea leads to fluid loss and may be life threatening particularly in young children and people who are malnourished or have impaired immunity [1]. This condition is the second leading cause of death in children every year. Many drugs are used as treatment for diarrhea; however, two or more of them are usually required for the treatment; thus their continuous use causes bacterial resistance and the subsequent loss of antibacterial efficacy [2, 3]. Natural products are an alternative for obtaining bioactive compounds with antidiarrheal activity [4]. Since immemorial times the entire orange fruit plant including fruits themselves, leaves, flowers, peels, and juice had been used for agriculture purposes, nutrition, and traditional medicine.* Citrus sinensis* (L.) Osbeck or sweet orange is consumed all over the world for being an excellent source of vitamin C and for its powerful antioxidant properties that build up the immune system. The orange fruit is composed of three sections, an external layer (peel), named flavedo, epicarp, or exocarp, a white portion below the exocarp, named albedo or mesocarp, and the innermost portion, the endocarp, that contains vesicles with juice and seeds [5, 6]. Orange is a good source of vitamins, minerals, and other nutrients. Many phytochemicals like limonoids, synephrine, hesperidin flavonoid, polyphenols, pectin, and enough folacin, calcium, potassium, thiamine, niacin, and magnesium are also present in it [7]. These biologically active compounds prevent arteriosclerosis, cancer, kidney stones, and stomach ulcers and cause a reduction in cholesterol levels and high blood pressure, promoting human health; thus, it possesses anti-inflammatory, antibacterial, larvicidal, and antifungal activity [7–11]. Reports suggest a high content of bioactive metabolites in leaves, flowers, and fruits, but few studies describe pharmacological effects of albedo and flavedo. The present study was undertaken in order to confirm the possible dual effect, spasmolytic and bactericide, of albedo and flavedo of two varieties of* C. sinensis* cultivated in Yucatán.

## 2. Materials and Methods

### 2.1. Chemicals and Drugs

Papaverine HCl, dimethyl sulfoxide (DMSO), and amikacin were purchased from Sigma-Aldrich Co. (St. Louis, MO, USA). Ethylic ether, n-hexane, and methanol were purchased from High-Purity Co. (Monterrey, NL, Mexico) ACS grade. Stock solutions of extracts were made using distilled water and freshly prepared the same day of experimentation.

### 2.2. Plant Material

Fruits of* Citrus sinensis* (L.) Osbeck National and Valencian varieties were collected in a crop local in Akil, Yucatán, México (20°14.9′N and 98°20.1′W) in December 2009. Plant material was authenticated by Salvador Flores Guido, PhD, from the Botany Department of Facultad de Veterinaria y Zootecnia of Universidad Autónoma de Yucatán (UADY). Voucher herbarium specimens were obtained and a specimen plant was deposited at UADY's herbarium “Alfredo Barrera Marín.”

### 2.3. Extraction

Firstly, mature fruits were washed with distilled water and, then, flavedo portion (peel) was separated mechanically, to get the albedo portion (white portion beneath the peel). The juice was extracted and the vegetal material was dried in a herbal desiccator at 50°C for three days to be grounded. Dried albedo (210 g) and flavedo (425 g) of* Citrus sinensis* National and Valencian varieties were successively extracted in a soxhlet apparatus first with* n*-hexane and then with methanol; the solutions were concentrated to dryness in a rotary evaporator (BUCHI, RII, Switzerland) at 45°C. Extraction yields (%, dry mass) were for hexanic extract of albedo National variety (HAN, 17.4%), methanolic extract of albedo National variety (MAN, 40.5%), hexanic extract of flavedo National variety (HFN, 6.1%), methanolic extract of flavedo National variety (MFN, 25.0%), hexanic extract of albedo Valencian variety (HAV, 17.9%), methanolic extract of albedo Valencian variety (MAV, 38.9%), hexanic extract of flavedo Valencian variety (HFV, 3.0%), and methanolic extract of flavedo Valencian variety (MFV, 26.2%). To carry out the experiments, all extracts were dissolved in a mixture of water: DMSO. Final concentration of DMSO inside the organ chamber never exceeded 0.1%.

### 2.4. Antimicrobial Activity

#### 2.4.1. Bacterial Cultures

The microorganisms used in the present investigation included reference strains from American Type Culture Collection (ATCC),* Escherichia coli* (ATCC 128),* Salmonella enterica *(ATCC 14028),* Salmonella enteritidis* (ATCC 22177), and* Salmonella choleraesuis* (ATCC 10708). The bacterial stock cultures were incubated for 24 h at 37°C on nutrient agar. Stocks cultures were retained at −20°C to use.

#### 2.4.2. Antibacterial Activity by the Method of Microdilution Plate Method

The eight organic extracts (HAN, MAN, HFN, MFN, HAV, MAV, HFV, and MFV) of* Citrus sinensis* (L.) Osbeck National and Valencian varieties were evaluated for antimicrobial activity against* Escherichia coli*,* Salmonella enterica*,* Salmonella enteritidis,* and* Salmonella choleraesuis* using the microdilution minimum inhibitory concentration (MIC) and minimum bacterial concentration (MBC) assays [12, 13]. The MBC was defined as the lowest recorded organic extract concentration of the MIC wells in which bacteria failed to grow. All procedures were performed so as to ensure sterility.

The eight organic extracts were diluted to a concentration of 1000 *μ*g/mL with DMSO as diluent. The microtiter plates were prepared adding 100 *μ*L of sterile nutrient broth into each well. Thereafter, the organic extracts and the positive control (Amikacin 12.5 *μ*g/mL) were added at a volume of 100 *μ*L. The organic extracts were serially diluted to reach concentrations of 1000, 500, 250, 125, 62.5, 31.2, 15.6, 7.8, 3.9, 1.9, 0.97, and 0.48 *μ*g/mL. Negative control was also included (nutrient broth with DMSO).

Finally, 100 *μ*L of inoculum (at 0.5 McFarland) was added to each well and the plates were incubated for 24 h at 37 ± 1°C. After incubation, 100 *μ*L of INT (2-(4-iodophenyl)-3-(4-nitrophenyl)-5-phenyltetrazolium chloride) at 0.02% was added to the reaction mixture, and then it was incubated at 37 ± 1°C in orbital shaking for 30 minutes. The interaction of the microorganisms (when viable) with INT gives rise to a color change from colorless to a reddish-pink color. The wells with the lowest dilution changes observed were considered as the MIC for these tested samples.

### 2.5. *Ex Vivo* Pharmacological Assay

#### 2.5.1. Animals

Healthy male Wistar rats were used and maintained under standard laboratory conditions with free access to food and water. All animal procedures were conducted in accordance with our Federal Regulations for Animal Experimentation and Care [14] and approved by the Institutional Animal Care and Use Committee. All experiments were carried out using six animals per group. All animals of the study were sacrificed by cervical dislocation.

#### 2.5.2. General Procedures

Rats (250–300 g body weight) were killed and abdominal dissection was carried out to extract the ileum. It was cleaned from excrement and adjacent and connective tissue and then cut into strips 2 cm long. Then, the tissue sections were assembled using stainless steel hooks under an optimal tension in chambers at 37°C containing Krebs-Henseleit solution (KHS; composition, mM: NaCl, 119; KCl, 4.6; KH_2_PO_4_, 1.2; MgSO_4_, 1.2; CaCl_2_, 1.5; NaHCO_3_, 20; and glucose 11.4; pH, 7.4) constantly bubbled with an O_2_ : CO_2_ (95 : 5) mixture. Changes in tension were recorded by force transducers Grass-FT03 (Astromed, West Warwick, RI, USA) connected to analyzer MP150 (BIOPAC 4.1 Instruments, Santa Barbara, CA, USA) as described previously by Estrada-Soto et al., 2010 [15].

#### 2.5.3. Rat Ileum Assay

Tissue segments (≈2 cm) were placed in organ baths containing 14 mL of KHS. All tissues were assembled with stainless steel hooks under an optimal tension of 1 g in organ baths with KH solution. After equilibration (15 min), a 10 min control period was recorded. The eight organic extracts (HAN, MAN, HFN, MFN, HAV, MAV, HFV, and MFV), positive control (Papaverine; phosphodiesterase inhibitor), and vehicle (DMSO 1%) were added to the bath in a volume of 100 *μ*L. Subsequently, cumulative concentration-response curves were obtained for each tissue segment with half-logarithm unit increments (Papaverine: 0.97→100 *μ*g/mL and extracts: 9.7→1000 *μ*g/mL). The effect of organic extracts and positive control on spontaneous contraction of ileum rings was determined by comparing the mean of the muscular tone and frequency inscribed by tissue contractions before and after addition of the test materials. Muscular tone was calculated from the tracings using Acknowledge Software (BIOPAC 4.1).

### 2.6. Data Analysis

The experimental results are expressed as mean of five experiments ± standard error of mean (SEM). Concentration responses curves (CRC) were plotted, and experimental data in the CRC were adjusted using the fit-sigmoidal (Hill equation) in the program Microcal™ Origin 8.6 (Microcal Software Inc., USA). Statistical analysis was conducted using one-way ANOVA, followed by Tukey's* post hoc* test. *p* < 0.05 was considered to imply significance of the pharmacological effects in the experiments.

### 2.7. Chromatographic Fingerprint Analysis

All the extracts were derivatized with boron trifluoride-methanol (BTF/MeOH) for gas chromatography analyses. 5 mg of each extract was added to 10 mL of BTF/MeOH and the mixture was heated under reflux for 20 min, and then the solutions were partitioned with 5 mL of n-hexane. 1 *μ*L of the hexane solution (5 mg/mL) containing the extract was injected in split mode (50 : 1 ratio by 1 min) in a gas chromatograph (Agilent Technologies 6890N, USA) equipped with a mass selective detector (5973Network, Agilent Technologies, USA) and a fused silica capillary column (J&W GC columns, USA) of 30 m × 0.25 mm × 0.25 *μ*m coated with cross-linked 5% phenyl-95% methyl polysiloxane. High purity (>99.999%) helium was used as carrier gas, at 0.8 mL/min with constant pressure. The oven temperature was programmed, 45°C for 3 min, and then increased 4°C/min to 250°C and stood by for 5 min; then a last increase of 20°C/min to 325°C took place, with a total time of 80 min for the analysis of each extract.

## 3. Results

*Citrus sinensis* (L.) O., commonly known as “sweet orange,” has been used for hundreds of years for its medicinal and nutritional properties [7].* C. sinensis* fruits are a main source of important phytochemical nutrients and for a long time they have been valued for their wholesome nutritious and antioxidant properties. Bactericide activity of flavedo portion has been investigated previously but albedo (mesocarp) is less investigated.

In Yucatán, México, two varieties of* C. sinensis *L. (Osbeck) are known. Their albedo and flavedo were subject to extraction process exhaustively and subsequently were evaluated for bactericidal activity and* ex vivo* spasmolytic effect. Additionally, chromatographic fingerprints of each extract were obtained and helped to correlate the pharmacological activity with the presence of secondary metabolites.

### 3.1. Antibacterial Activities

The antibacterial activities of the eight organic extracts (HAN, MAN, HFN, MFN, HAV, MAV, HFV, and MFV) of* Citrus sinensis* (L. Osbeck) National and Valencian varieties were studied in different concentrations (1000, 500, 250, 125, 62.5, 31.2, 15.6, 7.8, 3.9, 1.9, 0.97, and 0.48 *μ*g/mL) against four bacterial strains (*E. coli*,* S. enterica*,* S. enteritidis,* and* S. choleraesuis*). The results of the antibacterial activities are presented in Table 1. The extracts obtained from* Citrus sinensis* (L. Osbeck) National variety presented significant activity. The HFN had antimicrobial activity against* S. enteritidis* presenting MIC values of 1000 *μ*g/mL and MAN had activity against* S. choleraesuis* also with a MIC of 1000 *μ*g/mL. Regarding the extracts of* Citrus sinensis* (L. Osbeck) Valencian variety, only MAV had activity against* S. enteritidis* with a MIC of 1000 *μ*g/mL. The MBC was not determined for any of the strains tested as the required concentrations of the extracts were above the concentrations examined.

### 3.2. Spasmolytic Activity

**Figure 1:**
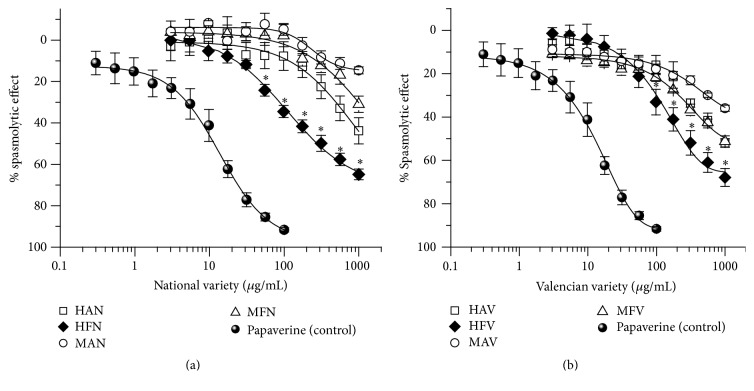
Concentration-response cumulative curves for spasmolytic activity of extracts of (a) National variety and (b) Valencian variety of* Citrus sinensis* albedo and flavedo on spontaneous contraction of ileum rat strips. Values are expressed as the percentage of inhibition of contractile responses calculated as the mean ± SEM from six animals, ^*∗*^*p* < 0.05.

Figure 1(a) shows concentration-response curves (CRC) of spasmolytic effect of* Citrus sinensis* National variety extracts (HAN, MAN, HFN, and MFN). The CRC of hexanic extract of flavedo (HFN) is significantly shifted to the left when compared to HAN, MAN, and MFN. HFN was more efficient (*E*_max_** = ***64.87 ± 3.04%)* and potent* (EC*_*50*_* = 300 μg/mL)* than HAN (*E*_max_ = 43.80 ± 6.32%; CE_50_ = ND), MAN (*E*_max_ = 14.62 ± 1.69%; CE50 = ND), and MFN (*E*_max_ = 31.01 ± 3.92%; CE_50_ = ND). On the other hand, Figure 1(b) shows CRC of spasmolytic effect of* C. sinensis* Valencian variety extracts (HAV, MAV, HFV, MFV). Again, the hexanic extract of flavedo (HFV) was more efficient (*E*_max_** = ***68.91 ± 4.14%)* and potent (EC_50_** = ***287.46 μg/mL)* compared with HAV (*E*_max_ = 51.98 ± 1.98%; CE_50_ = ND), MAV (*E*_max_ = 35.98 ± 1.42%; CE_50_ = ND), and MFV (*E*_max_ = 51.28 ± 2.59%; CE_50_ = ND) and it was also more powerful than HFN (National variety). All extracts of Valencian variety were more efficient that National variety as shown in Table 2. The evaluated extracts were neither more powerful nor more effective than Papaverine (phosphodiesterase inhibitor), used as positive control (*E*_max_ = 95%).

### 3.3. Fingerprint Chromatogram Analysis of Extracts

**Figure 2:**
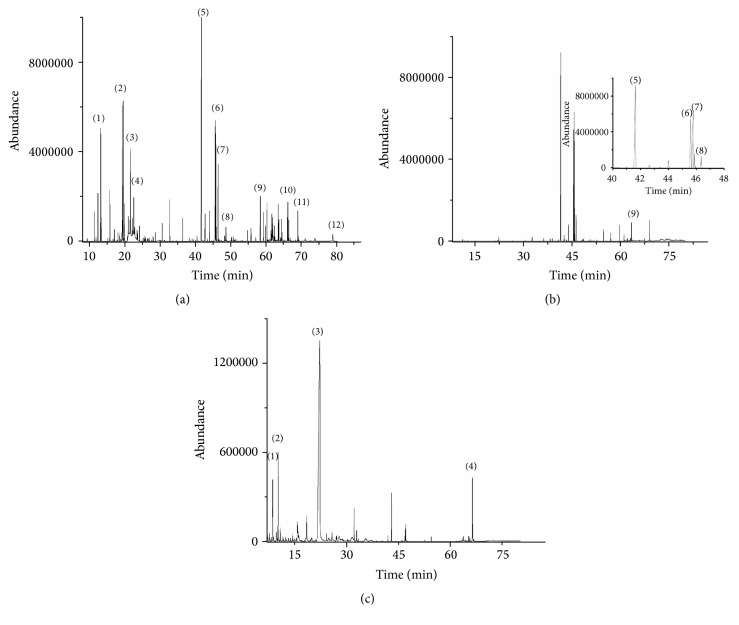
Comparative fingerprint chromatograms: (a) hexanic flavedo fraction, (b) hexanic albedo fraction, and (c) methanolic fraction for albedo and flavedo.

Many compounds eluted from capillary gas chromatography in each extract, so the chromatograms were divided into three sections. The first one includes chemical compounds with the lowest molecular weights (MW) as monoterpenes and sesquiterpenes; the second part consists of compounds with middle MW, mainly fatty acids between C14 to C18, and the last section consists of compounds with the highest MW containing triterpenoidic molecules. According to the spasmolytic effect and antibacterial activity of hexanic flavedo extracts from both varieties were significantly more efficient and potent on spasmolytic effect than other extracts. We compared fingerprints of hexanic extracts. Chromatographic fingerprint analysis of HFV and HFN suggests the presence of monoterpenes (8.1%; 38.2%), fatty acids (86.7%; 54.2%), and triterpenic (5.2%; 7.6%) compounds, respectively. The comparison of chromatographic fingerprints of Valencian (HFV) and National (HFN) hexanic flavedo extracts showed similar signals in the chromatogram (Figure 2(a)).

The monoterpenes (1) 1,3,8-p-methatriene, (2) D-limonene, (3) dehydrocymene, and (4) thujol were eluted in the first section (low MW) around minutes 10 to 25. In the second section (middle MW, 30 to 45 min) four main fatty acids were found: (5) palmitic, (6) linoleic, (7) oleic and (8), stearic. In the last section (high MW, 50 to 80 min) four signals were identified as triterpenoids: (9) *β*-sitosterol acetate, (10) *β*-sitosterol, (11) stigmastan-3-5-diene, and the last one (12) *β*-tocopherol.

The fingerprint analysis from hexanic albedo extracts (HAN and HAV) only showed a few signals in the second section of the chromatogram (Figure 2(b)). These signals were identified as the same fatty acids found in flavedo extracts; however, two more signals were identified in the last section corresponding to *β*-Sitosterol and Stigmastan-3-5-diene, only representing 2% of the total area of the chromatogram. The albedo extracts fingerprints (HAN and HAV) were significantly different from flavedo extracts, especially in the first section, which corresponded to small molecules with monoterpenic structure. The fingerprints of HAV and HAN (albedo extracts) that were less efficient on the* ex vivo* assay revealed monoterpenes (1.9%; 2.2%), fatty acids (97.3%; 96.5%), and triterpenoids (0.8%; 1.3%).

On the other hand, albedo (MAN and MAV) and flavedo (MFN and MFV) methanolic extracts (Figure 2(c)) showed similar signals. In these extracts (MAN, MAV, MFN, and MFV), the main signals were oxidative forms for carbohydrates such as (1) furfural, (2) furfural acetone, and (3) furfuraldehyde and the last signal was identified as (4) *β*-sitosterol.

## 4. Discussion

The methanolic extracts of both varieties showed higher yields in the extraction process compared to hexanic extracts. It has been described that metabolic content is greater in medicinal plants extracts obtained with solvents of medium to high polarity such as methanol [16]. Waxes and essential oils are among the phytochemicals which have been extracted from orange peel and they showed antimicrobial activity.

Chanthaphon et al., 2008, evaluated the antimicrobial activity of essential oils of kaffir lime, lime, pomelo, acidless orange, Chugun orange, and Nech kumquat [17], obtaining a MIC >2.25 mg/mL for* Salmonella sp.* and* E. coli.* Similar data is reported by Ou et al., 2015, where antibacterial activities of cold-pressed and distilled essential oils of* C. paradise* and* C. grandis *(L.) Osbeck resulted in a MIC 20 mg/mL for* Salmonella sp.* and* E. coli *[18]. Compared with our results we obtained a lower MIC 1000 *μ*g/mL for HFN, MAN, and MAV on* S. enteritidis* and* S. choleraesuis*. On the other hand, hexanic flavedos of both varieties showed significant spasmolytic effect (>60%) on ileum strips isolated from rat when compared to hexanic albedos and methanolic extracts.

Chromatographic fingerprint analysis of HFV and HFN suggests the presence of monoterpenes, fatty acids, and triterpenic compounds in higher proportion with respect to HAV and HAN (albedo extracts) and it is related to pharmacological activity. Flavedo's extract of national and Valencian varieties showed an efficient spasmolytic effect, possibly related to triterpenic or monoterpene compounds, since they are in greater quantity with respect to hexanic extracts of albedo (HAV and HAN). The antibacterial activity is only related to the amount of monoterpenes since the flavedo hexanic extract of national variety (38.2% of monoterpenes) was more active than flavedo hexanic extract of Valencian variety (8.1% of monoterpenes). Antimicrobial properties, anti-inflammatory activity, antiulcer activity, and antifungal activity has been reported in triterpenic compounds [19]. Also Prissadova et al., 2015, report smooth muscle relaxation of ursolic acid due to reducing the Ca^2+^ influx [20]. Monoterpenes such as 1,3,8-p-methatriene, d-limonene, dehydro-cymene, and thujol may be responsible for spasmolytic effect. Chemical, medical, and pharmacological literature suggests that citrus peel essential oils can be successfully used in many aspects of health care [21]. Several essential oils are reported to exhibit spasmolytic activity [22–25]. De Sousa et al., 2008, report spasmolytic effect of monoterpene (−)-carvone. Furthermore, (+)-limonene was efficient in contraction induced by 60 mM of KCl solution [26]. Besides Santos et al., 2011, reported vasorelaxant effect of p-cymene [25]. Methanolic extract showed antibacterial activity; the main constituent in the MAN and MAV was furfural, which has been reported to have antimicrobial activity [27].

## 5. Conclusion

*Citrus sinensis *(L.) Osbeck National variety had significative spasmolytic* ex vivo* effect and antibacterial activity due to presence of monoterpenic and triterpenic compounds in flavedo portion. This species represents an important source for obtaining bioactive compounds with therapeutic potential in the treatment of infectious diarrhea.

## Figures and Tables

**Table 1:** Minimum inhibitory concentration (MIC) and bactericidal (MBC) of hexane and methanolic extracts evaluated against strains of bacteria; the values are concentrations (*µ*g/mL).

Microorganism								
Extract	*Escherichia coli*		*Salmonella enterica*		*Salmonella enteritidis*		*Salmonella choleraesuis*	
	MIC	MBC	MIC	MBC	MIC	MBC	MIC	MBC
HAN	>1000	>1000	>1000	>1000	>1000	>1000	>1000	>1000
MAN	>1000	>1000	>1000	>1000	>1000	>1000	*1000*	>1000
HFN	>1000	>1000	>1000	>1000	*1000*	>1000	>1000	>1000
MFN	>1000	>1000	>1000	>1000	>1000	>1000	>1000	>1000
HAV	>1000	>1000	>1000	>1000	>1000	>1000	>1000	>1000
MAV	>1000	>1000	>1000	>1000	*1000*	>1000	>1000	>1000
HFV	>1000	>1000	>1000	>1000	>1000	>1000	>1000	>1000
MFV	>1000	>1000	>1000	>1000	>1000	>1000	>1000	>1000
AK	12.5	ND	12.5	ND	12.5	ND	12.5	ND

HAN: hexanic albedo of *C. sinensis* var. National; MAN: methanolic albedo of *C. sinensis* var. National; HFN: hexanic flavedo of *C. sinensis* var. National; MFN: methanolic flavedo of *C. sinensis* var. National; HAV: hexanic albedo of *C. sinensis* var. Valencian; MAV: methanolic albedo of *C. sinensis* var. Valencian; HFV: hexanic flavedo of *C. sinensis* var. Valencian; MFV: methanolic flavedo of *C. sinensis* var. Valencian; AK: Amikacin.

**Table 2:** Spasmolytic effect on rat ileum trips of two varieties of *Citrus sinensis* L. Osbeck.

National variety extracts	*E*_max_ (%)	EC_50_ (*µ*g/mL)	Valencian variety extracts	*E*_max_ (%)	EC_50_ (*µ*g/mL)
HAN	43.80 ± 6.32	ND	HAV	51.98 ± 1.98	ND
MAN	14.62 ± 1.69	ND	MAV	35.98 ± 1.42	ND
HFN	*64.87 ± 3.04*	300.31	HFV	*68.91 ± 4.14*	287.46
MFN	31.01 ± 3.92	ND	MFV	51.28 ± 2.59	ND

Papaverine (positive control): *E*_max_ = 90% and EC_50_ = 12.44 *µ*g/mL.

*E*_max_: maximum effect; EC_50_: effective concentration medium.

ND: Undetermined.
